# Influence of the Operator`s Experience, Working Time, and Working Position on the Quality of the Margin Width: In Vitro Study

**DOI:** 10.3390/medicina59020244

**Published:** 2023-01-27

**Authors:** Kinga Mária Jánosi, Diana Cerghizan, Zsigmond Rétyi, Alpár Kovács, Andrea Szász, Izabella Mureșan, Aurița Ioana Albu, Liana Georgiana Hănțoiu

**Affiliations:** 1 Faculty of Dental Medicine, George Emil Palade University of Medicine, Pharmacy, Science, and Technology of Targu Mures, 38 Gh. Marinescu Str., 540142 Targu Mures, Romania; 2 Independent Researcher, SC Fusion Dental Clinic SRL, 520089 Sfantu Gheorghe, Romania; 3 Independent Researcher, SC Maxdent Office SRL, 540501 Targu Mures, Romania

**Keywords:** heavy chamfer, tooth preparation, prosthodontics

## Abstract

*Background and Objectives*: Appropriate tooth preparation is mandatory to obtain a perfect marginal fit of fixed restorations. The heavy chamfer is the most commonly used finish line, especially for minimally invasive tooth preparation. The aim of the study was to compare the width of the finish line obtained during tooth preparation performed by experienced (university lecturers) and inexperienced persons (dental students) in different working times and positions. *Materials and Methods*: Forty left upper-second molars were prepared on the simulator by each participant, totalizing 160 prepared teeth. A new round-end tapered diamond was used to obtain the 0.5 mm width of the heavy chamfer. The prepared teeth were photographed using a Canon D5300 camera with a macro lens attached to a tripod. The measurements were made with the Image-Pro Insight software selecting the same eight reference points. From these points, perpendicular lines were drawn above the finish line to the axial walls and the distance between the chamfer’s outer edge and the axial wall’s inner edge was measured. GraphPad Instat and NCSS Dowson Edition software were used. The statistical significance was set at *p* < 0.05. The mean (M) and standard deviation (SD) were calculated. The used tests: one sample t-test, ANOVA test, and Tukey–Kramer Multiple Comparisons Test. *Results*: Statistically significant differences were obtained according to the experience of the participant, preparation time, patient’s position, and the chamfer width on the prepared tooth different surfaces. *Conclusions:* Daytime or weeklong tiredness and patient position do not affect the width of the heavy chamfer prepared by experienced and inexperienced persons. The experience and the operator’s working position influence the width of the prepared finish line.

## 1. Introduction

Proper tooth preparation is mandatory for the perfect marginal fit of fixed restorations. Inadequate preparation seems responsible for early failures such as caries, endodontic and periodontal complications [1]. This is due to the accumulation of the bacterial plaque at the microscopic opening at margins of the crowns [2]. The accuracy of clinical procedures during the tooth preparation and the manufacturing techniques of the restorations can condition the dimension of the marginal gap [3]. According to McLean and Von Fraunhofer, the clinically acceptable marginal gap is under 120 μm [4]. Aminian and Brunton have developed indications about the finish line preparation to avoid stress, improve the aesthetics, minimize the surface roughness, and eliminate the stress zones at this level [5]. The chamfer and the heavy chamfer have different indications; the recommended finish line for metal crowns is chamfer, for full ceramic crowns is heavy chamfer [6,7]. No scientific studies approve that the chamfer finish line is better than other finish lines [8]. For aesthetic ceramic restorations, the performed preparation should be minimally invasive. A ceramic thickness of at least 0.5 mm and preparation without exposing dentine are advantageous for adhesive cementation [9,10]. Enamel thickness varies at the different levels of teeth’s clinical crown surface: 0.3–0.5 mm cervical, 0.6–1 mm in the middle third, and 1.0–2.1 mm in the incisal third [11], and the minimally invasive preparations must be performed respecting these standards.

The study aimed to compare the width of the heavy chamfer prepared by experienced and inexperienced persons at different working times (different days of the week, various stages of the day) and the patient’s position (sitting and supine position).

Hypothesis: Daytime or weeklong tiredness and patient position do not affect the width of the chamfer prepared by experienced or inexperienced persons. There are no statistically significant differences between the width of the finish line and half the diameter of the diamond bur used for its preparation.

## 2. Materials and Methods

Two dental students from last year, t1 (female) and t2 (male), and two university lecturers from the University of Medicine and Pharmacy Targu Mures G. E. Palade, Faculty of Dental Medicine, Department of Fixed Prosthodontics, T1 and T2 (females), performed the preparations. Student t1 was trained by lecturer T1, and student t2 was trained by lecturer T2. The two instructors did the theoretical training of the participants following the same rules regarding the principles of tooth preparation with a heavy chamfer finish line. The participants learned about the techniques and instruments used during the preparation sessions and the ergonomic working positions for the sitting and supine patient positions. The practical training was carried out over a week. On the first and second days of the practical training, the students prepared an upper left second molar in the sitting position of the patient, similar to a study conducted by Won et al. [12], in which the preparations were performed on upper left premolars and upper left second molars in a random position and home position with ergonomically stable posture. T1 and t2 learned how to: prepare the occlusal surface morphologically by placing orientation grooves; avoid undercuts by maintaining the diamond bur parallel to the tooth axis; obtain a minimal taper close to 6°; to place equigingival the finish line. They prepared another molar in the supine patient position in the following two days. The preparations were conducted in both patient positions on the last training day. Each participant performed the tooth preparations with high-speed handpieces.

The sample size was calculated based on the standard deviation (SD) and mean (M) from a similar study by Lee and Choi [13] using a web-based sample size calculator. In their study [13], a total of one hundred prepared teeth by five operators were assessed regarding the chamfer width. Parameters: SD was 0.0908, M was 0.6283, the alpha level was set at 0.05, and the power of the test was set at 0.8. Based on the parameters, an estimated sample size of 58 teeth was obtained. An average value of the number of teeth prepared during one week of clinical activity of the two experienced persons was calculated, resulting 38 prepared teeth. Dividing the obtained value by working days resulted in eight prepared teeth/day/operator.

The desired finish line for the preparations was the equigingival heavy chamfer, which is used currently for minimally invasive preparations in the case of ceramic restorations. The preparation depth was adjusted to the minimum wall thickness of the ceramics in the lateral area (0.5 mm) [11]. According to Kasem et al. and Nakamura et al., the 0.5 mm margin width can be considered the ideal conservative thickness for zirconia and zirconia-reinforced glass-ceramic systems. Their recorded fracture resistance values of these crowns were superior to the maximum mastication force of humans (850 N) [14,15].

The artificial teeth were fixed in standardized positions in Planmeca simulators (Planmeca Oy, Helsinki, Finland) in the Simulation Center of the University of Medicine and Pharmacy Targu Mures G. E. Palade, Faculty of Dental Medicine. All the participants in this study prepared four teeth with the patient in a sitting position and four in a supine position every day of the week at the same time: Monday morning, Tuesday evening, Wednesday morning, Thursday evening, and Friday morning. A total number of 160 teeth were prepared. The preparations were performed from the operators sitting positions, in the 9–11 o’clock area according to ISO Standard 11226: “Ergonomics-Evaluation of static working postures”.

A new round-end tapered 016 diamond with a standard 3° taper was used to perform the preparations and obtain the 0.5 mm width of the heavy chamfer finish line. The active end of the diamond had a diameter of 1.1 mm. Each diamond bur was only used for one preparation. Only one half the diameter of the diamond bur was used to prepare the chamfer’s proper width to prevent unsupported enamel being left at the margins. Precise control and correct orientation of the diamond bur are mandatory to obtain the desired shape and width of the finish line and the proper taper. For this purpose, the diamond bur was maintained parallel to the tooth axis as well. Tilting away from the tooth will create undercuts (opposing axial preparation walls will diverge in an occlusal direction), and tilting towards the tooth will result in an excessive convergence angle of the preparation.

**Figure 1:**
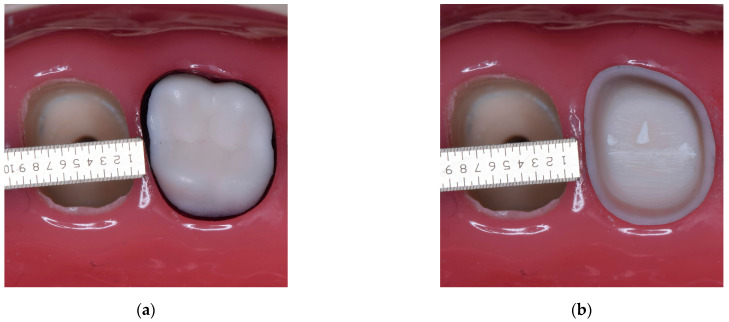
The upper second molar on the cast (**a**) The prosthetic equator, marked with a black pencil; (**b**) The calibration of the digital measurements.

The prepared teeth were removed and labeled after each preparation. They were divided into groups based on the operator, the working time, and patient position (sitting and supine). New unprepared teeth were positioned into the same spots. After every participant finished, the preparations and the casts supporting the artificial teeth were removed from the simulators. A cast with an unprepared second upper molar was placed on a survey table. The adjacent teeth (first upper molar and third upper molar) were removed from the cast. The prosthetic equator was marked with a black pencil at the maximum convexity of the tooth. The survey table was adjusted until the black line was visible from the occlusal view on the axial surfaces (Figure 1a). The survey table position was recorded as the reference position. The unprepared molar with the black line was removed from the cast. All the prepared teeth were placed in the reference position (the spot of the marked unprepared molar) and photographed twice using a Canon D5300 camera with a macro lens attached to a tripod. A ruler was placed parallel to the survey table, at the cervical area of the prepared tooth, at the same level as the finish line to calibrate the digital measurements (Figure 1b).

The photographs were selected and imported in the Image-Pro Insight software which was used for the measurements.

**Figure 2:**
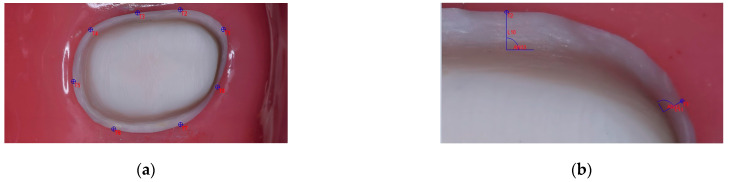
The measurements with the Image-Pro Insight software: (**a**) The eight reference points; (**b**) Measurements of the width of the chamfer at the reference points.

Eight reference points were selected (Figure 2a), and from these points perpendicular lines were drawn above the finish line to the axial walls. The distances between the chamfer’s outer edge and the axial wall’s inner edge were measured (Figure 2b) three times for each preparation by the same operator.

For statistical analysis of the data, GraphPad Instat and NCSS Dowson Edition software were used. The statistical significance was set at *p* < 0.05. The mean (M) and standard deviation (SD) were calculated. The used tests: one sample t-test, ANOVA test, and Tukey–Kramer Multiple Comparisons Test.

In a pilot study, the axial wall’s convergence was examined [16], considering the first preparations of each operator/day/patient position. The obtained values were considerably higher than the ideal values in the literature. Furthermore, the study concluded that the total convergence of the axial wall is independent of the operators’ experience or education level.

## 3. Results

The mean values (M) of the width of the daily prepared heavy chamfer and the standard deviation (SD) are represented in Table 1.

**Figure 3:**
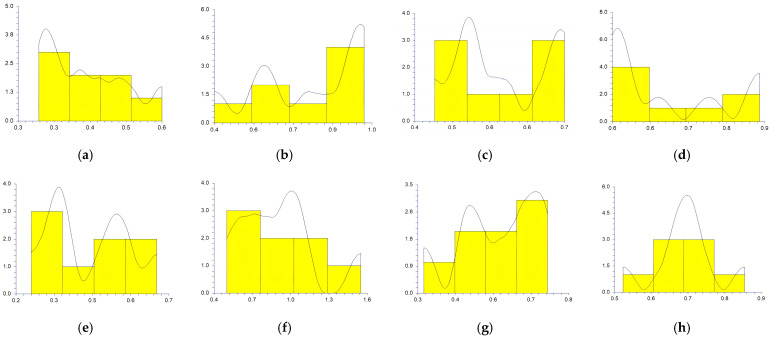
The chamfer width obtained at the reference points by all the participants Monday: (**a**) t1 sitting position; (**b**) t2 sitting position; (**c**) T1 sitting position; (**d**) T2 sitting position; (**e**) t1 supine position; (**f**) t2 supine position; (**g**) T1 supine position; (**h**) and T2 supine position.

**Figure 4:**
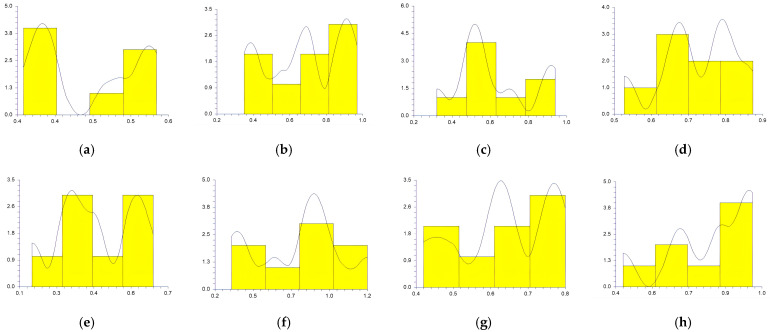
The chamfer width obtained at the reference points by all the participants Tuesday: (**a**) t1 sitting position; (**b**) t2 sitting position; (**c**) T1 sitting position; (**d**) T2 sitting position; (**e**) t1 supine position; (**f**) t2 supine position; (**g**) T1 supine position; (**h**) and T2 supine position.

**Figure 5:**
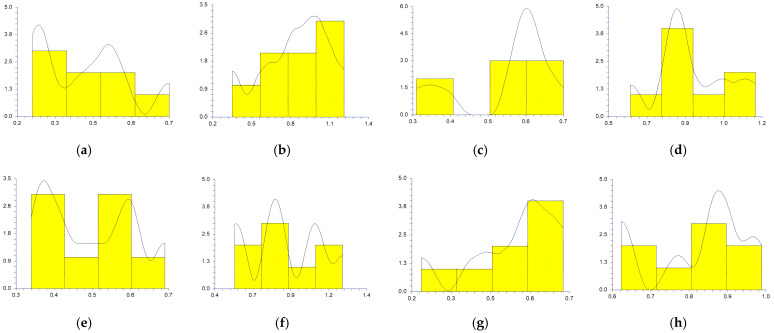
The chamfer width obtained at the reference points by all the participants Wednesday: (**a**) t1 sitting position; (**b**) t2 sitting position; (**c**) T1 sitting position; (**d**) T2 sitting position; (**e**) t1 supine position; (**f**) t2 supine position; (**g**) T1 supine position; (**h**) and T2 supine position.

**Figure 6:**
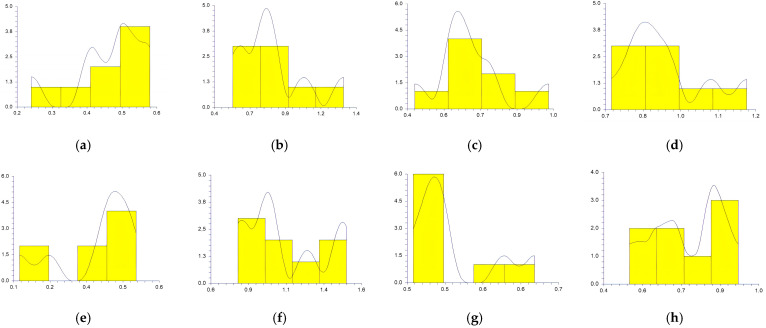
The chamfer width obtained at the reference points by all the participants Thursday: (**a**) t1 sitting position; (**b**) t2 sitting position; (**c**) T1 sitting position; (**d**) T2 sitting position; (**e**) t1 supine position; (**f**) t2 supine position; (**g**) T1 supine position; (**h**) and T2 supine position.

**Figure 7:**
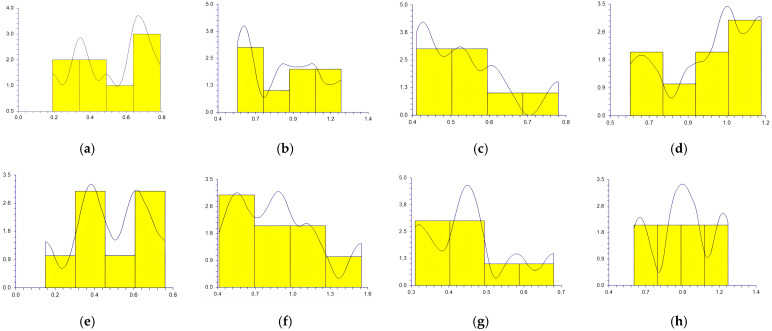
The chamfer width obtained at the reference points by all the participants Friday: (**a**) t1 sitting position; (**b**) t2 sitting position; (**c**) T1 sitting position; (**d**) T2 sitting position; (**e**) t1 supine position; (**f**) t2 supine position; (**g**) T1 supine position; (**h**) and T2 supine position.

The values obtained at the reference points by each participant in sitting and supine patient positions and workdays are represented in Figure 3, Figure 4, Figure 5, Figure 6 and Figure 7.

The mean values of the width of the chamfer prepared by all participants during the week are represented in Table 2.

The ANOVA test, *p* < 0.0001, showed an extremely significant variation among column means. The statistically significant results obtained by applying the Tukey–Kramer multiple comparison test are represented in Table 3.

Tukey–Kramer multiple comparison test revealed that the results obtained by the t1 on the first day are statistically different from most of those obtained by the T2 and t2 on the third, fourth, and fifth day. The same result was obtained for the t1 for the second, third, fourth, and fifth day. Regardless of the day and working position, the results obtained by the T1 are statistically different from those obtained by the t2. The supine working position results obtained by the T1 are statistically different from those obtained by the T2 in the sitting working position. Regarding the working positions, the results obtained by each study participant showed no statistical difference between sitting and supine positions.

Concerning the difference between the width of the chamfer finish line and the size of half of the diameter of the diamond used (0.5 mm), the one-sample t-test was applied. The results are presented in Table 4.

## 4. Discussion

Practitioners’ preferred method for tooth preparation margin design is the chamfer or heavy chamfer finish line [17,18]. It offers optimal marginal bulk for the restorations. Sadid-Zadeh et al. evaluated 392 STL files of posterior preparations for monolithic zirconia crowns and 82% presented a chamfer finish line [19]. In a research conducted by Shankar et al., 84% of 100 laboratory casts prepared for anterior all-ceramic crowns presented a radial shoulder finish line [20]. 

The fracture resistance of all-ceramic restorations, especially in the case of posterior crowns is a controversial topic in the research literature. According to Camille Haddad and Kathy Azzi, the marginal design of the abutment significantly affects the resistance of these aesthetic restorations [21]. Beuer et al. reported that the shoulder margin provided a higher fracture resistance than the deep chamfer and chamfer margins [22]. Sadan et al., in their study, found that both shoulder and chamfer finish lines are adequate for densely sintered alumina and zirconia restorations [23]. According to Alzahrani et al., the heavy chamfer margin could increase the breaking load of monolithic zirconia crowns [24].

Comparing the chamfer and the heavy chamfer finish line, Jalalian et al. demonstrated a higher fracture resistance for the heavy chamfer margins than the chamfer margins. The heavy chamfer finish line is recommended for posterior aesthetic restorations since it improves the biomechanical performance of posterior single zirconia crowns [25]. In an article-type review performed by Camille Haddad and Kathy Azzi, the conclusions showed that the monolithic zirconia crowns with knife-edge margins displayed superior fracture resistance at maximum occlusal forces compared to other types of finish lines [21].

For tooth preparation with a chamfer/heavy chamfer finish line the torpedo diamond, the round-end tapered diamond and the round-end taper with guide pin diamond can be used. The selection of a specific diamond bur is not scientifically supported [26]. According to Hooper, the most frequently used diamond bur in dental education is the torpedo-shaped diamond [27]. Boening et al. [28] and Mansueto et al. [29] demonstrated that it was much easier for dental students to prepare the finish line with round-end diamond burs than the torpedo diamond burs. In a study conducted by Măroiu et al., they used cylindrical shape diamond burs and rounded-tip Arkansas stones to prepare the tooth for ceramics [30]. Siegel and von Fraunhofer demonstrated that the efficiency of the rotary instruments is conditioned by the amount of dentinal debris on the diamond surface resulting from the tooth preparation [31]. Repeated use of rotary cutting instruments decreases their cutting efficiency [32]. For this reason, in our study a new round-end tapered diamond bur was used for each molar preparation contradicting Seymour’s results which highlighted that using new diamond bur will result in a wider finish line [1].

In a study conducted by SG Mihali et al., using minimal invasive preparation is essential in adhesive dentistry. The authors observed no failures for veneers with a maximum thickness of 0.5 mm [33]. Rammersberg et al. showed that a minimally invasive 0.5 mm width of the chamfer would result in good stability for posterior metal-free Artglass crowns cemented by adhesive technique [34]. According to Pan et al., for deciduous tooth preparation, a 0.4–0.6 mm shoulder and chamfer finish lines are recommended to ensure material strength and preserve the restoration’s marginal integrity [35]. Our results show wide variations regarding the obtained width of the heavy chamfer (Table 1, Figure 3, Figure 4, Figure 5, Figure 6 and Figure 7). The T1 participant approached the ideal width on the third working day in the sitting position of the patient (0.50125 mm) (Table 1). Considering the mean values obtained by all the participants in the study, the determined 0.5 mm value was best approximated by the t1 participant (0.45 mm), followed by the values of T1, T2, and t2 (Table 2). The values achieved by the t1 participant are less than 0.5 mm for most preparations (Table 1 and Figure 3, Figure 4, Figure 5, Figure 6 and Figure 7) contradicting the minimum necessary width for preventing the fracture of posterior zirconia crowns [17,18]. The two experienced participants, T1 and T2, obtained the most stable values (Figure 3, Figure 4, Figure 5, Figure 6 and Figure 7) due to their higher experience levels. Extremely significant differences can be found in the results obtained by T1 and T2 in different patient positions (Table 3). The finish lines prepared by the T2 participant are considerably wider than those prepared by the T1 person for each preparation regardless of the day and working position. The students obtained similar results to their instructors; t1 prepared less invasive than t2 (Table 1, Figure 3, Figure 4, Figure 5, Figure 6 and Figure 7). A study was performed by examining STL (Standard Tessellation Language) files of dental students from the records of the tooth preparations for metal-ceramic crowns. It was observed that the majority of the finish line’s width was between 0.5 and 1 mm, the maximum was approximately 2 mm and the minimum was 0 mm [36]. The present study’s results are similar, as observed in Table 1.

Siegel and von Fraunhofer demonstrated in the case study [31] that most dental practitioners prefer to remove the tooth structure in excess during the preparation of the tooth. Our specialists (T1, T2) removed more dental tissues than 0.5 mm (Table 2, Figure 3, Figure 4, Figure 5, Figure 6 and Figure 7). A minimum thickness of 0.5 mm is essential to prevent warpage of zirconia restorations [37]. Hey et al., in their study, found that most of the inexperienced participants removed too much rather than too little tooth structure [38], as in the case of t2.

Diamond burs with rounded ends and inactive guide pins allow a more suitable preparation at the margins without increasing the risk of hard tissue damage or other mechanical or thermal side effects [28]. According to Xiaoxiang Xu et al. [39], standardized practical training can improve dental students tooth preparation skills. Individual training must be conducted based on different tooth, patient, and bur positions. Improvement in the dental students’ performance was observed in another study, in which the preparations were performed with used diamond burs [29].

In the present study, it was demonstrated that the working position does not influence the quality of the tooth preparation. These results agree with In-Jae Won et al., which concluded in a study conducted in 2011 that the working position does not influence the marginal width of the preparation [12]. Lee and Choi obtained different results. They demonstrated that the removed tooth structure at the margins is higher in the random operator position than in ergonomic position [13].

According to LeBlanc, virtual training and in vitro preparations are viable methods of developing dental practitioners’ skills [40]. In disagreement, in a study by Stoilov et al., it was observed that students using digital assessment tools had significant problems recognizing and correcting their preparation deficiencies [41]. Haptic and traditional simulators could be used equally to train dental students in acquiring skills and understanding relevant concepts about tooth preparation [42].

Digital technology [43,44] and the use of magnification [45] can be helpful not only in minimal tooth preparation, but also in achieving the proper postures according to the principles of ergonomics [46]. Carpentier et al. and Aldosari concluded that magnification significantly improves the posture but not the quality of tooth preparation [47,48]. In contrast, Eichenberger et al. showed that magnification devices improved the precision of tooth preparation in a simulated clinical condition. Very significant differences were noted when a microscope was used for the tooth preparation followed by preparation using loupes; precision was lowest without magnification aids, with prescription glasses if needed (*p* < 0.0001) for both indirect and direct vision (*p* < 0.05) [45].

Combining digital and traditional education and evaluation methods are essential for effective dental education and highly quoted preparation activity. Traditional methods do not allow an objective evaluation of the quality of tooth preparation. Therefore, implementing digital techniques is mandatory in preclinical and clinical education [49]. Using simulators and magnification in preclinical training improves the achievement of ergonomic positions. Intraoral scanners allow for objective evaluation, self-evaluation, and their use to improve the quality of future restorations. 

The most common investigative methods available to practitioners [30] for evaluation require further investigations using magnification, digital scanners, and optical microscopy. The expansion of the number of participants and the variety of the prepared teeth is also the subject of future research concerning the limits of the minimally invasive preparation for CAD/CAM technologies.

Limitations of our study:

- In vivo conditions could not be reproduced perfectly. Even the best simulators cannot reproduce the intraoral conditions perfectly (visibility, muscle and tongue tonicity, saliva, and the patient’s movements).
- The low number of participants—a higher number could give different results depending on skill and experience.
- The use of one type of tooth for the preparation—different types of teeth from different regions could lead to different results and improve the complexity of the study.
- Subjective human assessment of the measurements. Using total digital working methods (scanning and digital analysis) can improve the accuracy of the measurements.

## 5. Conclusions

Within the limitations of this in vitro study we can conclude that daytime or weeklong tiredness and patient position do not affect the width of the chamfer prepared by experienced and inexperienced persons.

The experience and the operator’s working position influence the width of the finish line. The preparation depth can be conditioned by the methodology of the instruction (work position), and the instructor’s work style.

## Figures and Tables

**Table 1:** Descriptive statistics of the obtained values.

		Position	Mean	Standard Deviation	Minimum	Maximum
1 Day	t 1	Sitting	0.4175	0.1062006	0.3000	0.6000
		Supine	0.4375	0.1410927	0.2500	0.6600
	T 1	Sitting	0.57625	9.999107E-02	0.4400	0.7000
		Supine	0.565	0.1393864	0.3200	0.7300
	t 2	Sitting	0.765	0.2173214	0.4000	0.9700
		Supine	0.90875	0.3267344	0.5000	1.550
	T 2	Sitting	0.6625	0.1263499	0.5500	0.8400
		Supine	0.6675	8.172253E-02	0.5200	0.8100
2 Day	t 1	Sitting	0.4675	8.972179E-02	0.3600	0.5800
		Supine	0.41125	0.1664278	0.1500	0.6400
	T 1	Sitting	0.6175	0.2141928	0.3200	0.9400
		Supine	0.63625	0.1340509	0.4200	0.8000
	t 2	Sitting	0.68125	0.2269322	0.3500	0.9700
		Supine	0.745	0.3009034	0.3100	1.200
	T 2	Sitting	0.7025	0.1248714	0.4800	0.8700
		Supine	0.78375	0.1863896	0.4300	0.9600
3 Day	t 1	Sitting	0.425	0.155747	0.2500	0.7000
		Supine	0.495	0.1247855	0.3400	0.6900
	T 1	Sitting	0.50125	0.1341042	0.2600	0.6500
		Supine	0.5225	0.1492601	0.2300	0.6800
	t 2	Sitting	0.83625	0.277434	0.3500	1.210
		Supine	0.855	0.2514529	0.5300	1.240
	T 2	Sitting	0.8825	0.1805349	0.6000	1.170
		Supine	0.79375	0.1204678	0.6200	0.9400
4 Day	t 1	Sitting	0.46625	0.1100568	0.2400	0.5800
		Supine	0.385	0.1404076	0.1200	0.5200
	T 1	Sitting	0.65875	0.1582437	0.4300	0.9800
		Supine	0.525	7.111359E-02	0.4600	0.6600
	t 2	Sitting	0.81	0.2532644	0.5300	1.310
		Supine	1.09	0.2751104	0.7800	1.500
	T 2	Sitting	0.89875	0.1462324	0.7200	1.170
		Supine	0.725	0.1457983	0.5000	0.9200
5 Day	t 1	Sitting	0.52125	0.2118246	0.1900	0.7900
		Supine	0.48125	0.1988134	0.1500	0.7600
	T 1	Sitting	0.5375	0.1239528	0.4100	0.7800
		Supine	0.45875	0.122991	0.3100	0.6800
	t 2	Sitting	0.83125	0.2511367	0.5300	1.220
		Supine	0.8825	0.3702027	0.4100	1.550
	T 2	Sitting	0.94625	0.2178425	0.3100	0.6800
		Supine	0.9	0.2200649	0.5700	1.210

**Table 2:** Mean values of the width of the prepared chamfer.

Participants	Width of the Chamfer (Mean Values)	Differences
t 1	0.450000	-
t 2	0.840500	+
T 1	0.559125	+
T 2	0.79625	+

**Table 3:** Tukey–Kramer multiple comparison test–extremely significant differences.

Day	Operator	Position	Operator	Position	Difference
1	t 1	Sitting	t 2	Supine	−0.4913 ***
	t 1	Supine	t 2	Supine	−0.4713 **
3	t 1	Sitting	T 2	Sitting	−0.4575 **
	t 1	Sitting	t 2	Supine	−0.4300 **
	t 1	Sitting	t 2	Sitting	−0.4113 *
	T 1	Sitting	T 2	Sitting	−0.3813 *
	t 1	Supine	T 2	Sitting	−0.3875 *
4	t 1	Sitting	t 2	Supine	−0.6238 ***
	t 1	Supine	T 2	Sitting	−0.5138 ***
	t 1	Supine	t 2	Supine	−0.7050 ***
	T 1	Supine	t 2	Supine	−0.5650 ***
	t 1	Sitting	T 2	Sitting	−0.4325 **
	t 1	Supine	t 2	Sitting	−0.4250 **
	T 1	Sitting	t 2F	Supine	−0.4313 **
5	t 1	Sitting	T 2	Sitting	−0.4250 **
	t 1	Sitting	T 2	Supine	−0.3788 *
	t 1	Supine	T 2	Sitting	−0.4650 **
	t 1	Supine	T 2	Supine	−0.4188 *
	t 1	Supine	t 2	Supine	−0.4013 *
	T 1	Sitting	T 2	Sitting	−0.4088 *
	T 1	Supine	T 2	Sitting	−0.4875 ***
	T 1	Supine	T 2	Supine	−0.4413 **
	T1	Supine	t 2	Supine	−0.4238 **

*** extremely significant, ** very significant, * significant.

**Table 4:** One-sample *t*-test results.

Day	Position	Operator	*p* Value
1	Sitting	t1	0.063994
		t2	0.010706
		T1	0.067921
		T2	0.008313
	Supine	t1	0.250468
		t2	0.009490
		T1	0.228685
		T2	0.000665
2	Sitting	t1	0.339675
		t2	0.058413
		T1	0.164699
		T2	0.002523
	Supine	t1	0.175214
		t2	0.054753
		T1	0.023827
		T2	0.003541
3	Sitting	t1	0.215394
		t2	0.011014
		T1	0.979703
		T2	0.000546
	Supine	t1	0.912950
		t2	0.005235
		T1	0.682645
		T2	0.000232
4	Sitting	t1	0.414484
		t2	0.010518
		T1	0.025135
		T2	0.000115
	Supine	t1	0.053662
		t2	0.000508
		T1	0.353181
		T2	0.003294
5	Sitting	t1	0.784815
		t2	0.007352
		T1	0.420487
		T2	0.000668
	Supine	t1	0.797351
		t2	0.022264
		T1	0.374402
		T2	0.001337

## Data Availability

The dataset analyzed during the study are available from the first author on request.

## References

[1] Seymour K.G., Cherukara G.P., Samarawickrama D.Y., Zou L. (2008). Consistency of labial finish line preparation for metal ceramic crowns: An investigation of a new bur. J. Prosthodont..

[2] Sirajuddin S., Narasappa K.M., Gundapaneni V., Chungkham S., Walikar A.S. (2015). Iatrogenic damage to periodontium by restorative treatment procedures: An overview. Open Dent. J..

[3] Sarda A.S., Bedia S.V. (2021). Influence of manufacturing technique on marginal fit of cobalt chromium restorations: An in-vitro study. Indian J. Dent. Res..

[4] McLean J.W., Von F. (1971). The estimation of cement film thickness by an in vivo technique. Br. Dent. J..

[5] Aminian A., Brunton P.A. (2003). A comparison of the depths produced using three different tooth preparation techniques. J. Prosthet. Dent..

[6] Tsitrou E.A., Northeast S.E., van Noort R. (2007). Evaluation of the marginal fit of three margin designs of resin composite crowns using CAD/CAM. J. Dent..

[7] Beuer F., Edelhoff D., Gernet W., Naumann M. (2008). Effect of preparation angles on the precision of zirconia crown copings fabricated by CAD/CAM system. Dent. Mater. J..

[8] Valderhaug J. (1980). Periodontal conditions and carious lesions following the insertion of fixed prostheses: A 10-year follow up study. Int. Dent. J..

[9] Valderhaug J., Birkeland J.M. (1976). Periodontal conditions in patients 5 years following insertion of fixed prostheses. J. Oral Rehabil..

[10] Newsome P., Owen S. (2009). Improving your margins. J. Aesthetic Dent. Today.

[11] Goodacre C.J., Compagni W.V., Aquilino S.A. (2011). Tooth preparations for complete crowns: An art form based on scientific principles. J. Prosthet. Dent..

[12] Won I.J., Kwon K.R., Pae A.R., Choi D.G. (2011). An influence of operator’s posture on the shape of prepared tooth surfaces for fixed partial denture. J. Korean Acad. Prosthodont..

[13] Lee S.J., Choi D.G. (2001). The influence of home position (HP) and random position (RP) on the shape of prepared tooth surfaces-upper left 1st molar for full cast crown. J. Dent. Rehabil. Appl. Sci..

[14] Kasem A.T., Sakrana A.A., Ellayeh M., Özcan M. (2020). Evaluation of zirconia and zirconia-reinforced glass ceramic systems fabricated for minimal invasive preparations using a novel standardization method. J. Esthet. Restor. Dent..

[15] Nakamura K., Harada A., Inagaki R., Kanno T., Niwano Y., Milleding P., Örtengren U. (2015). Fracture resistance of monolithic zirconia molar crowns with reduced thickness. Acta Odontol. Scand..

[16] Cerghizan D., Crăciun A., Albu A., Baloș M., Jánosi K.M. (2020). In vitro study about the abutment axial wall’s convergence. Acta Stomatol. Marisiensis.

[17] Minyé H.M., Gilbert G.H., Litaker M.S., Mungia R., Meyerowitz C., Louis D.R., Slootsky A., Gordan V.V., McCracken M.S., National Dental PBRN Collaborative Group (2018). Preparation techniques used to make single-unit crowns: Findings from the national dental practice-based research network. J. Prosthodont..

[18] Jubhari E.H., Lesal E. (2021). Finish line for full coverage crown: A systematic review. J. Dentomaxillofac. Sci..

[19] Sadid-Zadeh R., Sahraoui H., Lawson B., Cox R. (2021). Assessment of tooth preparations submitted to dental laboratories for fabrication of monolithic zirconia crowns. Dent. J..

[20] Shankar S., Gounder R., Ganapathy D. (2020). Standards of teeth preparation for anterior all-ceramic crowns in private dental practice. Drug Invent. Today.

[21] Haddad C., Azzi K. (2022). Influence of the type and thickness of cervical margins on the strength of posterior monolithic zirconia crowns: A review. Eur. J. Gen. Dent..

[22] Beuer F., Aggstaller H., Edelhoff D., Gernet W. (2008). Effect of preparation design on the fracture resistance of zirconia crown copings. Dent. Mater. J..

[23] Sadan A., Blatz M.B., Lang B. (2005). Clinical considerations for densely sintered alumina and zirconia restorations: Part 1. Int. J. Periodontics Restor. Dent..

[24] Alzahrani A.M., Beyari A.M., Emam Z.N. (2018). The influence of the cervical finish line designs on the fracture resistance of CAD/CAM monolithic zirconia crowns: An in vitro study. Int. J. Health Sci. Res..

[25] Jalalian E., Rostami R., Atashkar B. (2011). Comparison of chamfer and deep chamfer preparation designs on the fracture resistance of zirconia core restorations. J. Dent. Res. Dent. Clin. Dent. Prospects.

[26] Siegel S.C., von Fraunhofer J.A. (1999). Dental burs what bur for which application? A survey of dental schools. J. Prosthodont..

[27] Hooper S.M., Huggett R., Foster L.V. (1993). Teaching veneer and crown margins in UK dental schools. Dent. Update.

[28] Boening K.W., Kaestner K.I., Luthardt R.G., Walter M.H. (2001). Burs with guide pins for standardized tooth preparation. Quintessence Int..

[29] Mansueto M.A., Abdulkarim H.A., Thabet W.R., Haney S.J. (2010). The chamfer finish line: Preclinical student performance using different bur designs. J. Dent. Educ..

[30] Măroiu A.-C., Sinescu C., Duma V.-F., Topală F., Jivănescu A., Popovici P.M., Tudor A., Romînu M. (2021). Micro-CT and microscopy study of internal and marginal gap to tooth surface of crenelated versus conventional dental indirect veneers. Medicina.

[31] Siegel S.C., von Fraunhofer J.A. (1999). Dental cutting with diamond burs: Heavy-handed or light-touch?. J. Prosthodont..

[32] Rotella M., Ercoli C., Funkenbusch P.D., Russell S., Feng C. (2014). Performance of single-use and multiuse diamond rotary cutting instruments with turbine and electric handpieces. J. Prosthet. Dent..

[33] Mihali S.G., Lolos D., Popa G., Tudor A., Bratu D.C. (2022). Retrospective long-term clinical outcome of feldspathic ceramic veneers. Materials.

[34] Rammelsberg P., Eickemeyer G., Erdelt K., Pospiech P. (2000). Fracture resistance of posterior metal-free polymer crowns. J. Prosthet. Dent..

[35] Pan C.-Y., Lan T.-H., Liu P.-H., Fu W.-R. (2020). Comparison of different cervical finish lines of all-ceramic crowns on primary molars in finite element analysis. Materials.

[36] Tiu J., Lin T., Al-Amleh B., Waddell J.N. (2016). Convergence angles and margin widths of tooth preparations by New Zealand dental students. J. Prosthet. Dent..

[37] Denry I., Kelly J.R. (2008). State of the art of zirconia for dental applications. Dent. Mater..

[38] Hey J., Schweyen R., Kupfer P., Beuer F. (2017). Influence of preparation design on the quality of tooth preparation in preclinical dental education. J. Dent. Sci..

[39] Xu X., Xie Q., Zhou Y., Wu L., Cao Y. (2020). Effect of a standardized training with digital evaluation on the improvement of prosthodontic faculty’s performance in crown preparation: A pre-post design. J. Prosthodont..

[40] LeBlanc V.R., Urbankova A., Hadavi F., Lichtenthal R.M. (2004). A preliminary study in using virtual reality to train dental students. J. Dent. Educ..

[41] Stoilov M., Trebess L., Klemmer M., Stark H., Enkling N., Kraus D. (2021). Comparison of digital self-assessment systems and faculty feedback for tooth preparation in a preclinical simulation. Int. J. Environ. Res. Public Health.

[42] San Diego J.P., Newton T.J., Sagoo A.K., Aston T.-A., Banerjee A., Quinn B.F.A., Cox M.J. (2022). Learning clinical skills using haptic vs. phantom head dental chair simulators in removal of artificial caries: Cluster-randomized trials with two cohorts’ cavity preparation. Dent. J..

[43] Valenti M., Schmitz J.H., Cortellini D., Valenti A., Canale A. (2021). A diagnostically and digitally driven tooth preparation protocol by using a patient monitoring tool with an intraoral scanner. J. Prosthet. Dent..

[44] Liu C.X., Gao J., Zhao Y.W., Fan L., Jia L.M., Hu N., Mei Z.Y., Dong B., Zhang Q.Q., Yu H.Y. (2020). Precise tooth preparation technique guided by 3D printing guide plate with quantitative hole. West China J. Stomatol..

[45] Eichenberger M., Biner N., Amato M., Lussi A., Perrin P. (2018). Effect of magnification on the precision of tooth preparation in dentistry. Oper. Dent..

[46] Yu H. (2022). Digital Guided Micro Prosthodontics.

[47] Carpentier M., Aubeux D., Armengol V., Pérez F., Prud’homme T., Gaudin A. (2019). The effect of magnification loupes on spontaneous posture change of dental students during preclinical restorative training. J. Dent. Educ..

[48] Aldosari M.A. (2021). Dental magnification loupes: An update of the evidence. J. Contemp. Dent. Pract..

[49] Güth J.F., Wallbach J., Stimmelmayr M., Gernet W., Beuer F., Edelhoff D. (2013). Computer-aided evaluation of preparations for CAD/CAM-fabricated all-ceramic crowns. Clin. Oral Investig..

